# Supplementary material for: Effectiveness and Safety of Ayurvedic Medicines in Type 2 Diabetes Mellitus Management: A Systematic Review and Meta-Analysis
Source: Front Pharmacol. 2022 Jun 8;13:821810. doi: 10.3389/fphar.2022.821810 (PMC9213670; doi:10.3389/fphar.2022.821810)
Supplement: Supplementary file 1 [file DataSheet1.zip › Supplementary Material/Forest and Funnel Plots/Plantago ovata/FBG.pdf]

Plantago ovata - FBG

Study	Intervention N	Intervention Mean	Intervention SD	Comparator N	Comparator Mean	Comparator SD	Mean Diff. with 95% CI	Weight (%)
Ziai 2005	21	155.60	43.53	15	216.20	97.99	-60.60 [-107.77, -13.43]	3.12
Feinglos 2013	26	186.53	25.23	8	215.05	26.11	-28.52 [-48.67, -8.37]	17.09
Abutair 2016	18	120.00	16.48	18	151.00	11.65	-31.00 [-40.32, -21.68]	79.79
**Overall**							-31.50 [-39.83, -23.17]	

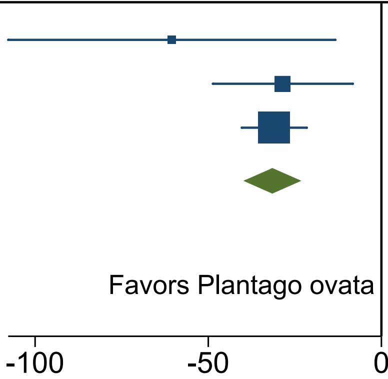

Heterogeneity: $\tau^2 = 0.00$, $I^2 = 0.00\%$, $H^2 = 1.00$

Test of $\theta_i = \theta_j$: Q(2) = 1.56, p = 0.46

Test of $\theta = 0$: z = -7.41, p = 0.00

Random-effects REML model
